# Fluid and Solute Intakes Show Minimal Association With Serum Sodium Levels in a Mixed ICU Population

**DOI:** 10.7759/cureus.37730

**Published:** 2023-04-17

**Authors:** John T Culhane, Divya Velury, Raymond I Okeke, Carl Freeman

**Affiliations:** 1 Surgery, Saint Louis University School of Medicine, Saint Louis, USA; 2 General Surgery, Saint Louis University School of Medicine, Saint Louis, USA; 3 General Surgery, SSM Health Saint Louis University Hospital, Saint Louis, USA; 4 Trauma, Saint Louis University School of Medicine, Saint Louis, USA

**Keywords:** trauma, electrolyte, sodium, fluid, hyponatremia

## Abstract

Background: Hyponatremia is common among hospital inpatients. It is generally due to excess free body water resulting from increased water intake and decreased water elimination due to underlying pathology and hormonal influence. However, supporting evidence is lacking for treating mild hyponatremia with fluid restriction. Our study examines the association between hyponatremia and fluid intake in acutely ill inpatients. We hypothesize that fluid intake is not closely associated with serum sodium (SNa).

Methods: We conducted a retrospective study of hyponatremia using the Multiparameter Intelligent Monitoring in Intensive Care (MIMIC) III dataset, a public ICU registry. We analyzed fluid, sodium, and potassium intake with a mixed model linear regression with SNa as the outcome for hyponatremic and non-hyponatremic patients and cumulative total input from one to seven days. In addition, we compared a group of patients receiving less than one liter of fluid per day to a group receiving more than one liter.

Results: The association of SNa with fluid intake was negative and statistically significant for most cumulative days of intake from one to seven for the total population and those with sporadic hyponatremia. For those with uniform hyponatremia, the negative association was significant for three and four days of cumulative input. The change in SNa was almost always less than 1 mmol/L of additional fluid intake across all groups. SNa for hyponatremic patients who received less than one liter of fluid per day were within one mmol/L of those who received more (p<0.001 for one, two, and seven cumulative intake days).

Conclusions: SNa is associated with a change of less than 1 mmol/L across a wide range of fluid and sodium intake in adult ICU patients. Patients who received less than one liter per day had SNa almost identical to those who received more. This suggests that SNa is not tightly coupled with fluid intake in the acutely ill population and that hormonal control of water elimination is the predominant mechanism. This might explain why the correction of hyponatremia by fluid restriction is often difficult.

## Introduction

Hyponatremia is the most common electrolyte disorder among hospital inpatients, with an estimated prevalence of 15-40% [1]. Hyponatremia is associated with a worse prognosis. Mortality, ICU admission, length of stay, and costs are all higher in hyponatremic patients; however, it is unclear whether the serum sodium (SNa) is a cause of morbidity or a marker of disease severity [2]. Despite the long-standing recognition and high prevalence of hyponatremia, the disorder remains a diagnostic and therapeutic challenge. Although new options for diagnosing and treating hyponatremia have appeared in recent years, the evidence base for therapy is still deficient [2]. Fluid restriction, in particular, lacks high-level evidence of clinical benefit. According to the latest American consensus guidelines for the management of hyponatremia, “Fluid restriction has traditionally been regarded as first-line therapy, despite the absence of an evidence-based rationale for its effectiveness” [3]. Our study examines the association between fluid intake and SNa in acutely ill patients. Considering the lack of evidence supporting the practice of fluid restriction to treat hyponatremia in acute illness, we hypothesize that SNa is not strongly associated with fluid volume intake, even at daily volumes less than one liter. The traditional management of hyponatremia with fluid restriction may not be practical in the acutely ill population.

## Materials and methods

We conducted a single-center, retrospective, observational study of hyponatremia using data from the Multiparameter Intelligent Monitoring in Intensive Care (MIMIC) III database. MIMIC is a publicly available registry of deidentified ICU data from multiple admissions over 11 years at Beth Israel Deaconess Medical Center. The population is a mixture of medical and surgical ICU patients. Although surgical patients may require more fluid than medical, we chose to include both to make the results more broadly applicable. This database is distinctive for its granularity, including details down to specific lab values and detailed recording of drug and fluid administration [4]. We selected patients 15 years of age and older and retrieved SNa for each day of admission. We averaged the values if more than one value was available per day. We reviewed all measured input sources to determine the amount of fluid, sodium, and potassium the patient received each day. Sources include maintenance intravenous (IV) fluid, IV infusions such as sedatives, bolus medications including electrolytes, the carrier solutions for IV piggyback drugs, total parenteral nutrition (TPN), enteral feeding formula, and oral intake.

We totaled electrolyte and fluid intake for specified days prior to each SNa measurement. We included only sequential days because data for missing days could affect results. We analyzed the association between SNa and fluid intake over the previous two, three, four, and seven days. Hyponatremia is defined as SNa<135 mmol/L [5]. If any of the daily SNa values within a series of days was less than 135, the hyponatremia was classified as sporadic. If all the values were less than 135, the hyponatremia was classified as uniform. Multivariate linear regression was performed with a mixed model to account for hierarchical data composed of repeat measures for individual patients. The outcome variable was SNa. The random effect parameter was the patient identifier. Fixed effect parameters included age, sex, fluid intake, sodium, and potassium intake. In addition, we identified comorbidities likely to affect SNa and included fixed-effect covariates. These include brain trauma, end-stage renal disease, chronic kidney disease, acute renal insufficiency, liver cirrhosis, congestive heart failure, hypertension, and diabetes mellitus. Finally, we identified patients receiving blood, colloid, and diuretics and included their status as fixed effect covariates in the model. We identified the group of patients with uniform hyponatremia who received an average of less than one liter of fluid per day, corresponding to a typical fluid restriction regimen. Then, we performed a propensity match based on the conditional probability of low fluid intake predicted by the same covariates used in the multivariate analysis. We used Student’s t-test to compare SNa for propensity-matched groups of patients who received less than one liter versus greater than one liter of fluid per day. Statistics were performed with Statistical Product and Service Solutions (SPSS) (IBM SPSS Statistics for Windows, Version 27.0, Armonk, NY).

## Results

A total of 37,754 patients were analyzed. The mean age was 64.1 years. Male patients made up 56.5% of the cohort. A majority of the patients (54.5%) were on the medicine service, and hypertension was the most prevalent ailment (46.7%) (Table 1). A total of 240,859 days of input/output were analyzed. There were 11,761 (31.1%) patients with at least one day of hyponatremia (SNa<135). There were 632 (1.7%) patients with at least one day of severe hyponatremia (SNa<125).

**Table 1:** Baseline characteristics

Baseline Characteristics		
	Mean	Median
Age	64.1	66
Service	n	%
Medical	20608	54.56
Surgical	14769	39.10
Trauma	2377	6.29
Total	37754	
Male Sex	21370	56.58
Brain Trauma	2510	6.65
End Stage Renal Disease	1078	2.85
Chronic Kidney Disease	1119	2.96
Acute Kidney Injury	9457	25.04
Liver Cirrhosis	2075	5.49
Congestive Heart Failure	10038	26.58
Hypertension	17626	46.67
Diabetes Mellitus	8912	23.60
Patients Receiving Blood Products	15276	40.44
Blood Products as percent of total fluid		16.68
Patients Receiving Colloids	6024	15.95
Colloid as percent of total fluid		3.85
Patients Receiving Diuretics	7423	19.65

The population was generally elderly with most patients over 65 years old. There was a slight preponderance of male patients. Medical and surgical services were fairly equally represented. Chronic disease was highly prevalent in this elderly cohort. Nearly one-half of patients were hypertensive. Congestive heart failure, diabetes mellitus, and acute kidney injury each affected approximately one-fourth of the patients.

Most of the fluid intake was composed of crystalloid, but blood products were another important source of volume at about one sixth of the total fluid intake. Colloid was less common at only about 4%. Diuretic use was included due to its potential effect on serum electrolytes. About 20% of patients received diuretics during the study period, thus for the majority electrolyte balance was not affected by pharmacologic diuresis.

The first column in Table 2 “All serum Na levels” refers to the entire population regardless of whether they experienced hyponatremia. The second column “Sporadic Hyponatremia” includes patients with at least one recorded sodium value below the lower reference range; that is, patients who were hyponatremic at any point during the study period. The third column “Uniform Hyponatremia” includes patients whose SNa was below normal on every measurement during the study period; that is, patients who were consistently hyponatremic. We defined these categories because patients who are hyponatremic may have a different response to fluid and electrolyte intake than those with normal sodium.

**Table 2:** Response of SNa to intake of fluid volume and sodium for acute inpatient population. SNa: serum sodium

	All Serum			Sporadic			Uniform	
	Na Levels			Hyponatremia			Hyponatremia	
	B	Sig		B	Sig		B	Sig
1 Cumulative Day								
Liters Total Fluid/Day	-0.067	<0.001		-0.152	<0.001		-0.012	0.814
Liters Free Fluid/Day	-0.062	<0.001		-0.093	<0.001		-0.077	0.360
Grams Na/Day	0.008	<0.001		0.017	<0.001		0.013	0.033
2 Cumulative Days								
Liters Total Fluid/Day	-0.087	<0.001		-0.114	<0.001		-0.061	0.212
Liters Free Fluid/Day	-0.057	<0.001		-0.047	0.046		-0.029	0.752
Grams Na/Day	0.008	<0.001		0.012	<0.001		0.010	0.075
3 Cumulative Days								
Liters Total Fluid/Day	-0.106	<0.001		-1.020	<0.001		-0.149	0.004
Liters Free Fluid/Day	-0.077	<0.001		-0.056	0.008		-0.152	0.197
Grams Na/Day	0.010	<0.001		0.010	<0.001		0.018	0.003
4 Cumulative Days								
Liters Total Fluid/Day	-0.112	<0.001		-0.090	<0.001		-0.154	0.005
Liters Free Fluid/Day	-0.098	<0.001		-0.073	<0.001		-0.320	0.028
Grams Na/Day	0.011	<0.001		0.009	<0.001		0.021	0.001
7 Cumulative Days								
Liters Total Fluid/Day	-0.083	<0.001		-0.040	0.020		-0.095	0.234
Liters Free Fluid/Day	-0.068	<0.001		-0.028	0.097		-0.352	0.082
Grams Na/Day	0.008	<0.001		0.003	0.111		0.011	0.200

B is the estimate of the slope of the mixed model regression line. This represents the change in mmol/L of SNa for each additional liter of fluid or gram of sodium per day. For example, take the values at the lower left corner of the table. For all patients in the cohort, after seven cumulative days of recorded fluid and sodium input, an additional liter of fluid intake per day is associated with an increase of SNa of 0.083 mmol/liter. The p-value is <0.001. This is a statistically significant, but minor association, less than one-tenth of one mmol/liter.

In Table 3, we classified the patients by the amount of fluid they received. The left columns of the table show the amount of fluid received by patients in the low intake group (<1000 ml/day) versus the high intake group (>1000 ml/day). The right columns show the average SNa in the low versus high intake groups.

**Table 3:** Serum sodium (SNa) following fluid intake <1000 ml/day versus >1000 ml/day for the uniform hyponatremia group. N refers to the number of patients in both the low-intake and high-intake groups averaged over the specified number of cumulative days. The propensity match identifies equal numbers of low-intake patients and controls making the sample size equal between exposure groups.

Cumulative		Average Daily Fluid (ml)			Serum Sodium (mEq/L)
Days	n	Low Intake	High Intake	Sig	Low Intake
1	2338	682	1480.9	<0.001	131.8
2	1378	745.3	1444.2	<0.001	131.9
3	918	785.2	1408	<0.001	132
4	733	808.7	1396.2	<0.001	132
7	401	851.7	1409.5	<0.001	132

## Discussion

Hyponatremia (SNa<135 mmol/L) is the most common electrolyte disorder treated in clinical practice. It is a chronic and acute illness present in 15-20% of emergency admissions [6]. Our study showed an even higher prevalence of 31.1% in a mixed ICU population. Hyponatremia can be challenging to understand and treat because it is not only an electrolyte disorder but also one of water balance. Therefore, one cannot simply treat the sodium deficiency by giving more solute without addressing the cause of disordered water homeostasis. Hyponatremia is a heterogeneous disorder with various causes and features in different populations. Categorization typically begins with a clinical assessment of the volume status, which can be difficult to perform accurately [7].

Further subcategories are hypotonic versus non-hypotonic hyponatremia. Non-hypotonic is due to the presence of other solutes, such as glucose. It is rare, occurring mainly in severe hyperglycemia and renal failure, and is not the focus of our analysis. Euvolemic hypotonic hypovolemia is the most common subtype and is most commonly due to the syndrome of inappropriate antidiuretic hormone secretion (SIADH) [8]. SIADH results from inappropriately high levels of the hormone arginine vasopressin (AVP), which plays a central role in the renal regulation of water and indirectly of sodium [9]. Criteria for diagnosis of SIADH include low SNa along with clinical euvolemia, low serum osmolality, and elevated urine sodium and osmolality [10]. Complete biochemical workup of SIADH is only sometimes performed in clinical practice. Since diagnosis relies on the clinical assessment of euvolemia, it necessarily includes a clinical component. Clinicians may rely on a clinical diagnosis of SIADH for both patient care and scientific investigation. Hauptman et al. created a large registry of hyponatremia patients with entry criteria of SNa of <=130 and clinical euvolemia [11]. This registry subsequently formed the basis of multiple studies of hyponatremia [12-14].

Given the underlying endocrine cause of most hyponatremia, we sought to examine how solute and fluid intake interact with the hormonal state in ICU patients and what are the implications for treatment. Normally functioning kidneys can excrete free water or conversely concentrate urine sodium to excrete solute, thus conserving free water. This allows the body to maintain normal SNa values despite widely varying solute and volume intake. However, variations in fluid and sodium intake may exceed the ability of the kidney to adapt [15]. Some studies have measured the association between fluid intake and SNa in the acute setting. In a review of maintenance fluids in acutely ill patients, Moritz et al. reported that most hyponatremia is due to SIADH, but that the concentration and volume of fluid intake also play a role. They state that almost all studies of hospital-acquired hyponatremia show an association with hypotonic fluid administration [16]. Sim et al. found that volume balance in the ICU is associated with hyponatremia (19.4 mL/kg vs 11.5 mL/kg, P = 0.004). Their multivariate analysis confirmed the result with an OR of 1.004 for inclusion in the hyponatremia group, a statistically significant, but small increase [17].

The fluid does not have to be hypotonic to contribute to hyponatremia. A long-recognized phenomenon known as desalination occurs when the kidney excretes solute in urine at higher concentrations than the fluid that is infused. In this circumstance, even isotonic fluid infusions can lead to hyponatremia [18]. For this reason, we analyze the contribution of both the total volume and the free water component of fluid intake. Although fluid intake is the primary determinant of SNa levels, an association with solute intake has also been identified. McGreal et al. explain the mechanism by which sodium intake can influence hyponatremia [5].

Our study also showed an association between hyponatremia and intake of volume and sodium, which was significant but very small. These results are displayed in Table 2. The slope of the regression (B) reflects the change in SNa associated with varying fluid intake. Large changes in fluid intake are associated with very small changes in SNa - less than one mEq/L for each liter of variation in water intake. The table illustrates that SNa varies minimally with fluid intake even across wide ranges of fluid volume. Renal homeostasis tends to preserve SNa across varying fluid intake, but the treatment paradigm of fluid restriction for SIADH involves increasing SNa by reducing volume. Fluid restriction depends on limiting volume below the minimum necessary for the kidney to concentrate and excrete sodium, typically one liter per day. The volume restriction must be severe enough to overcome renal solute regulation in the presence of excess ADH. It must be extreme to work, which raises the question of practical difficulties.

American and European guidelines recommend hypertonic saline (typically 3% sodium chloride) for acute or symptomatic hyponatremia [3,10]. According to a 2021 review, in modern practice, for most other cases of mild-to-moderate hyponatremia, especially those due to SIADH, fluid restriction is the first-line treatment [19]. Fluid restriction is inexpensive, easy to administer, unlikely to cause overcorrection, and is based on a plausible physiologic mechanism. Therefore, it remains a popular treatment option despite the lack of evidence of clinical benefit [20]. According to American guidelines, fluid restriction “has generally been the treatment of choice despite the almost complete lack of a supportive evidence base” [3]. A subsequent review of clinical practice guidelines regarding the diagnosis and treatment of hyponatremia found that 98% of guidelines were based on low and very low levels of evidence, and none were based on high levels [21].

In a 2016 review, Cuesta et al. stated that there have been no randomized controlled trials (RCTs) proving the efficacy or safety of fluid restriction in the clinical setting [8]. Since this review, three small, randomized trials have been published. Krisanapan et al. found that NaCl supplementation and furosemide provided no benefit over fluid restriction monotherapy, however, they did not compare fluid restriction versus no therapy [22]. An RCT in pediatric surgery patients showed that increased tonicity of fluid, but not fluid restriction reduced the incidence of hyponatremia [23]. Another RCT of fluid restriction to 1L/d in chronic hyponatremia resulted in an improvement of 3 mmol/L vs 1 mmol/L after three days (p=0.005). Subsequent therapy yielded no further improvement. Thus, a strict fluid restriction protocol provided a modest net increase of 2 mmol/L SNa in chronic hyponatremia [24]. Verbalis et al. conducted a study of the hyponatremia registry. Fluid restriction monotherapy was the treatment for 48% of patients and was associated with a Na increase of 1 mEq/L/day.

Fluid restriction alone failed to achieve prospectively defined Na improvement goals in >50% of cases, and 75% were still hyponatremic at discharge. Correction of hyponatremia was not associated with survival, suggesting that it is a marker of disease severity rather than an independent cause of mortality [14]. In an analysis of predictors of the efficacy of fluid restriction to treat hyponatremia, Winzeler et al. found that fluid restriction was ineffective in 41% of patients [25]. Thus, even with more recent data, high-level evidence of the efficacy of fluid restriction for hyponatremia is still lacking. Our study shows a minimal change in SNa across a wide range of daily fluid intake: less than 1 mmol/L change in SNa per liter of fluid intake. Despite the small absolute difference, most of the results achieved statistical significance. The large sample size and corresponding statistical significance indicate that the change in SNa with fluid intake is genuinely very small and is unlikely a result of type 2 error. Although the association of SNa with fluid intake is statistically significant, we believe it is not clinically important due to its small size.

Our findings are subject to some limitations. Strict diagnosis of SIADH requires measurement of urine and plasma osmolality, and urine sodium. We rely on a clinical diagnosis without extensive laboratory confirmatory tests. Fluid restriction must limit intake below a certain threshold to be effective. We addressed this by comparing a group that received liberal fluid intake with another that received less than one liter per day of fluid, consistent with a typical fluid restriction regimen. SNa following the lower intake was almost identical to the liberal fluid group, however, it is possible that more severe fluid restriction could be effective. Another possibility is that low SNa prompted clinicians to treat some of their hyponatremic patients with fluid restriction, thus low SNa could indirectly be a cause of low fluid intake. We address this possibility by analyzing a subgroup of patients who were uniformly hyponatremic throughout the period of study. The fluid restriction would likely be prescribed for all these patients; thus, the effects of selective fluid restriction should be minimized. Record keeping, especially intake and output, may not be accurate, which is an inherent limitation of retrospective data.

## Conclusions

SNa is almost unchanged across a wide range of fluid and sodium intake in acutely ill hospital inpatients. This includes volumes typically associated with a regimen of fluid restriction used to treat hyponatremia. We believe that this supports hormonally mediated water elimination over water intake as the primary determinant of SNa in the critically ill adult population. Fluid restriction is based on a sound physiologic mechanism, but the practical application has been challenging and strong evidence of efficacy has been lacking. Volume intake within the variation documented for this cohort of ICU patients showed minimal association with SNa. We consider it unlikely that deliberate restriction within these same parameters would have a greater effect. The resistance of SNa to variations in fluid volume suggests that treatment of hyponatremia with fluid restriction alone will be difficult in the acutely ill population. The practice is based on a model of renal function rather than empirical data. Withholding free water below the minimum that the kidney requires to excrete a solute load will eventually concentrate SNa, but our study helps explain why this is difficult to implement as a practical treatment protocol.
